# Some Smaller Hospitals and the Wounded

**Published:** 1914-11-28

**Authors:** 


### Some Smaller Hospitals and the Wounded.

The admission of the wounded, to the smaller hospitals especially, has led to the offer of accommodation by some smaller institutions under circumstances which a fuller appreciation of the necessity for providing only the very best and most hygienic accommodation for wounded warriors would have forbidden. Indeed, we hold distinctly that it is quite wrong for any hospital to offer to take in wounded warriors when the only plan the committee can evolve for their reception is to add, say, six additional beds to every ward containing twelve beds, and to arrange beds in the out-patient department without making provision to keep the portion of the out-patient buildings devoted to soldier patients distinct and separate from that where out-patients are continuously being seen every day. The War Office by its refusals has shown good judgment, and so saved the reputation of ambitious people associated with the management of institutions offering beds under the faulty conditions we refer to. The justification urged in favour of crowding existing wards is that by the admission of sailor or soldier patients when wounded into the additional beds referred to, no diminution has taken place in the means of treatment provided by the hospital for civilian patients. We would venture to point out that hygienically it is neither justifiable nor right for any hospital to increase the number of beds in its wards by 50 per cent., for such a step must entail new risks to both the civil and war patients that no experienced and knowledgeable administrator would suffer. Fortunately, so far as our observations extend, where overcrowding has been sanctioned, only slightly wounded patients have been sent.

